# Changes in physical performance and frailty-associated risk associated with participation in a community exercise programme: Findings from the ExWell cohort

**DOI:** 10.1016/j.tjfa.2026.100199

**Published:** 2026-10-05

**Authors:** Muireann O’Shea, Paul Doody, Noel McCaffrey, Aodhaghan O'Deasmhunaigh, Fiona Skelly, Lisa Kelly, Sean P Kennelly, Susan M Smith, Noel McCarthy

**Affiliations:** a Discipline of Public Health and Primary Care, Trinity College Dublin, Ireland; b ExWell Medical, Dublin, Ireland; c Physiotherapy, ExWell Medical, Dublin, Ireland; d Discipline of Public Health and Primary Care, Trinity College Dublin, ExWell Medical, Dublin, Ireland; e Institute of Memory and Cognition, Tallaght University Hospital, Dept of Medical Gerontology, School of Medicine, Trinity College Dublin, Ireland

**Keywords:** Frailty, Ageing, Exercise therapy

## Abstract

**Introduction:**

Exercise can improve physical function and reduce disability in older adults living with frailty. ExWell Medical is a national, community-based, medically supported group exercise program for people living with chronic conditions in Ireland.

**Study aims/objectives:**

We aimed to evaluate the longitudinal association between baseline frailty status and outcomes including gait speed in ExWell programme participants.

**Methods:**

This is a secondary data analysis of a prospective cohort study. All participants attending an ExWell Medical induction in the Dublin/Kildare regions January-September 2023 were invited to participate. At baseline, 12, and 24 weeks we collected data on socio-demographics; health behaviours; measures of physical function; patient-reported outcomes. The cohort were stratified by Clinical Frailty Scale category at baseline.

**Results:**

We recruited 1290 participants (mean age 69 (SD:10.9) years; 60% female. At baseline 15% of participants were classified as frail, 23% prefrail, 62% robust. Baseline frailty was associated with a higher chronic condition count, higher health service use, poorer scores on measures of physical function, EQVAS, EQ5D5L index scores. There was an inverse association between frailty and class attendance (p < 0.001). Attrition increased with increasing baseline frailty status (p = 0.006). Participants who were frail at baseline had improvements between baseline and 24-week reassessment in gait speed, timed up and go, six-minute time trial, EQVAS; prefrail participants in the sit to stand test, six-minute time trial, EQVAS, but no improvement in timed up and go. Robust participants recorded improvements in all measures.

**Conclusion:**

Participation in the ExWell programme was associated with improvements across frailty groups in community-dwelling adults living with chronic conditions.

## Highlights

• Participation in the ExWell programme was associated with improvements across frailty groups.
• Higher levels of frailty were associated with increased attrition rates.
• Focus should be placed on engaging and retaining more frail participants.
• Strategies to improve adherence and reduce attrition warrant further investigation.

## 1 Introduction

Frailty is a complex multidimensional geriatric syndrome characterised by a state of increased susceptibility to stressors. It is associated with an increased risk of adverse outcomes including falls, disability, hospitalisation, and mortality [1,2]. In Ireland, 12.7% of community-dwelling adults aged ≥ 50 years, and 21.5% aged ≥ 65 years are estimated to be living with frailty [3], which is comparable to international estimates [4]. Ireland, similar to most developed countries, has an ageing population, and the number of adults aged greater than 65 years is predicted to exceed one million by 2030 [5].

Exercise interventions have been shown to prevent and reverse frailty and pre-frailty among older adults [[6], [7], [8], [9], [10]]. Evidence suggests this only applies to interventions conducted within group settings, with mixed reports for single, condition-specific management programs [8]. Multicomponent exercise programs have been found to be more effective than resistance training alone [6], and are recommended in national and international guidance on physical activity for older adults [11,12]. Evidence suggests adherence to structured community-based exercise programmes is high [7,13].

There are numerous operational definitions for the classification of frailty [14], which are predominantly based on two principal models. The phenotypic model focuses on the identification of several physical traits such as weight loss, low energy and reduced physical activity [15]. In contrast, the cumulative deficit model focuses on the accumulation of multiple health deficits including chronic diseases [16,17]. Although the Fried frailty phenotype and the Frailty Index are among the most commonly used operational definitions, they are relatively time and data intensive and therefore somewhat difficult to implement at scale in the community setting. The Clinical Frailty Scale is a judgement-based frailty assessment tool that is easy to use, administered by trained clinicians, and has comparable predictive validity to the Fried frailty phenotype and Frailty Index [18]. Gait speed has also been proposed as a pragmatic proxy indicator of frailty in clinical and research settings [[19], [20], [21], [22], [23]] Gait speed less than 0.8m/s has been proposed as a simple proxy indicator of frailty in primary care setting [24,25]. In real-world clinical and service datasets, longitudinal measurement of frailty using comprehensive instruments is often not feasible, so objective physical performance measures, particularly gait speed, are frequently used as pragmatic markers of frailty risk and predictors of adverse outcomes.

Frailty is a dynamic condition commonly involving transitions between frailty states; with transition to increasing frailty more common than transition to decreasing frailty [2,26]. Transitions between frailty states in older adults are common but rates of transition from frail to non-frail states are low. Lower frailty prevalence in the younger older adult population presents opportunities for early identification and interventions to halt or reverse frailty progression [27].

The aim of this study was to examine the longitudinal association between baseline frailty status and health related outcomes for adults living with chronic conditions and multimorbidity attending an established community-based exercise rehabilitation programme.

## 2 Methods

### 2.1 Study design

This was a secondary analysis of data from a prospective cohort study of a community-based exercise rehabilitation programme (CBER) program. The main quantitative outcomes are reported in a separate publication [28] and qualitative analysis is in progress. The CBER programme (ExWell Medical) was funded by the Health Service Executive (HSE) in the Dublin and Kildare regions (Community Healthcare Organisation (CHO) 7). Ethical approval was obtained from Trinity College Dublin’s Research Ethics Committee (reference number 221002). We reported our findings in accordance with the Strengthening the Reporting of Observational Studies in Epidemiology (STROBE) checklist (Supplementary Data Appendix A) [29].

### 2.2 ExWell programme

ExWell Medical [30] is a medically designed CBER program for people living with chronic conditions in Ireland, referred by their healthcare practitioners. It involves twice weekly supervised group exercise classes conducted in community fitness centres. Classes involve a warm-up, aerobic, resistance, core stability and balance exercises, and a cool down. The standard class structure is based on stations and accommodates participants with various chronic conditions and different levels of functional ability (Supplementary Data Appendix D).

### 2.3 Setting and sampling

Potential participants referred to an ExWell class within the study area by a healthcare professional in primary or specialty care were contacted by an administrator, and invited to attend an induction session delivered by a medical doctor. All patients who were referred to ExWell and attended an induction in the study area between 30 January and 22 September 2023 were invited to participate. Potential participants were provided with a link to a participant information leaflet prior to attending their scheduled induction session. Participation in the study was voluntary and those declining to join the research project continued to participate in the ExWell program. All participants provided written informed consent.

### 2.4 Population

Eligible participants were aged ≥ 18 years, living with at least one diagnosed chronic condition, and referred to ExWell by a healthcare professional. We excluded those unable to participate in the program due to disabling physical conditions (e.g., uncontrolled cardiac conditions) or with mental health conditions, cognitive impairments, or language difficulties that restricted communication or capacity to consent.

### 2.5 Data collection

Data for the main cohort study were collected at baseline (induction session), 12-, and 24 weeks (reassessment sessions) by trained members of the ExWell team. This paper focuses on baseline and 24-week outcome assessments. Blinding of outcome assessors was not possible. Participants were invited to complete assessments regardless of how many classes they had attended. Study participants no longer attending the ExWell program were invited by phone call from the study team to return for a specific follow-up session at 24 weeks post-induction to complete the patient questionnaire and physical outcome measures. For those unable to attend, telephone interviews were conducted to complete patient reported outcome measures (PROMs), and capture reasons for non-attendance, where possible. Participant recruitment, retention, and individual participant class attendance were also recorded.

**Fig. 1:**
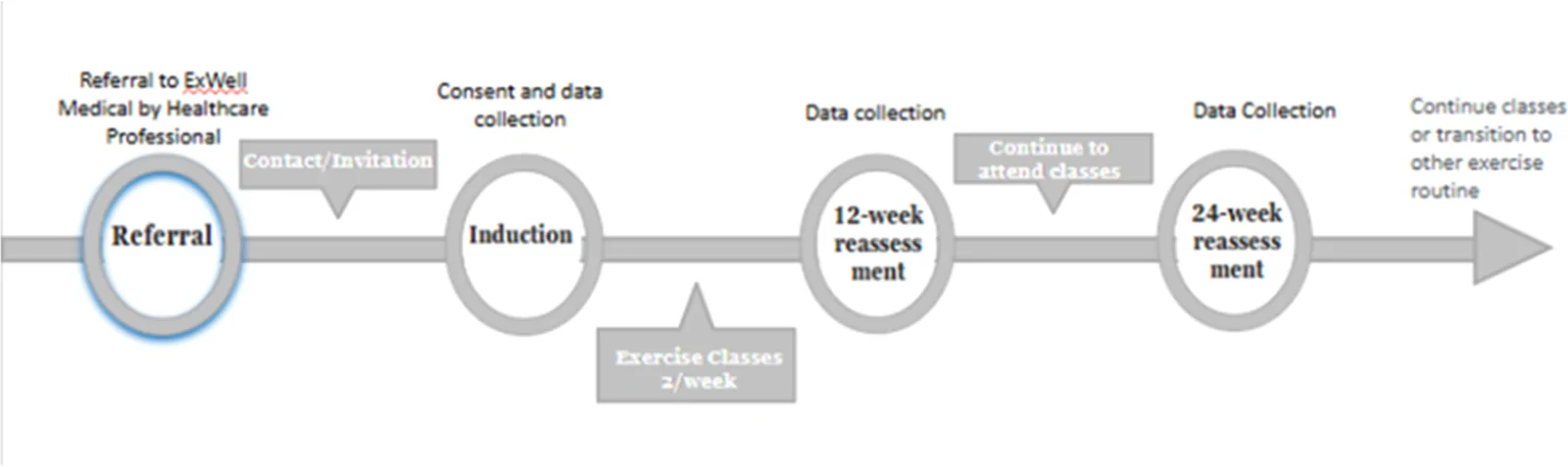
Intended flow of study participants.

### 2.6 Outcome measures

For this secondary analysis, we identified in advance which outcomes we would consider based on their hypothesised association with frailty. We collected data regarding demographics, health, health behaviour (Supplementary Data Appendix E), measures of physical function (Supplementary Data Appendix B), and patient reported outcome measures (Box 1). Frailty status at baseline was assessed using the Clinical Frailty Scale (CFS), a validated multidimensional measure of frailty incorporating clinical judgement across functional and cognitive domains [18]. At follow-up, a repeat multidimensional frailty assessment was not available. Therefore, gait speed was used as an objective outcome measure of physical performance and as a proxy indicator of frailty-associated risk. Gait speed is a well-established predictor of adverse outcomes and forms a core component of frailty phenotypes.

Box 1 **Participant characteristic and study outcome measures**

• Age, sex, marital status
• Health behaviours (smoking and alcohol use)
• Number of chronic conditions
• Number of healthcare services attended
• Clinical Frailty Status
• Physical measures:
  ○ Six-minute walk test (m)
  ○ Gait speed test(m/s)
  ○ Timed up and go (m/s)
  ○ Five sit to stand test (*sec*)
• Patient reported outcome measures
  ○ Health related quality of life using the EQVAS and EQ5D5L index score [28,29]

### 2.7 Data analysis

Descriptive statistics were used to summarise the baseline demographic details of enrolled participants. Demographic details and selected measures of physical function and patient reported outcome measures (PROMs) were categorized by frailty status determined by the Clinical Frailty Scale at baseline. Due to small numbers in some of the Clinical Frailty Scale categories, the categories were grouped into three groups to assist with analysis, presentation, and interpretation of results. The ‘Very Fit’, ‘Well’ and ‘Managing Well’ groups were grouped under ‘Robust’. The ‘Prefrail’ group remained unchanged. ‘Mildly Frail’, ‘Moderately Frail’, ‘Severely Frail’, and ‘Terminally Ill’ groups were combined to form the ‘Frail’ group’.

Change in mean scores from baseline to 24-week reassessment for select outcome measures were analysed by Clinical Frailty Scale category. Differences between frailty groups were assessed using ANOVA, Kruskal Wallis, or chi square tests. The primary analysis of the impact of the program at the pre-defined study endpoint (24 weeks) was conducted as a complete case analysis and included the participants with data available at both time points

Descriptive statistics were utilised to assess frailty risk pre and post intervention using gait speed as a proxy indicator of frailty-associated risk. Participants were stratified into groupings of frail, prefrail, and robust based on gait speed results (<0.8m/s frail, 0.8–1.2 m/s prefrail, >1.2m/s robust) [19,31,25].

Programme attrition by frailty status group was assessed using Pearson chi square test.

Multivariable logistic regression was used to assess whether frailty status predicted program dropout. Linear regression was used to assess the impact of class attendance on gait speed at 24 weeks. All statistical analyses were performed in STATA [32].

## 3 Results

Between January 30th and September 22nd 2023, 1290 participants were enrolled (mean age 69 (SD:10.90) years; 60% female). The mean number of healthcare services attended was 1.6 (SD:1.44) and mean number of chronic conditions was 3.3 (SD:2.17).

The Clinical Frailty Scale was conducted for all participants at induction to the ExWell program. Table 1 presents baseline data for study participants by Clinical Frailty Scale status.

**Table 1:** Participant demographics, outcomes measures, and class attendance by Clinical Frailty Scale categories.

				Robust (n, SD/%)	Prefrail (n, SD/%)	Frail (n, SD/%)	p-value
				n = 668	n = 250	n = 166	
**Age**				69 (10.6)	71 (9.9)	72 (11.9)	p < 0.001
**Gender**		female		390 (58%)	161 (64%)	96 (58%)	p = 0.22
		male		278 (42%)	89 (36%)	70 (42%)	
**Marital Status**		unmarried		254 (38%)	99 (40%)	66 (40%)	p = 0.91
		married		400 (60%)	143 (57%)	96 (58%	
		unknown		14 (2%)	7 (3%)	3 (2%)	
				**Robust (mean, SD)**	**Prefrail (mean, SD)**	**Frail (mean, SD)**	**p value**
**No of chronic diseases**			3.12 (1.96)		3.74 (2.19)	3.96(2.69)	p < 0.001
**Number of health care services attended**			1.34 (1.12)		1.72 (1.47)	2.51 (2.04)	p < 0.001
**Number of classes attended**			17.59 (12.48)		15.95 (12.13)	12.93 (11.57)	p < 0.001
**Gait speed (m/s)**			1.24(0.25)		1.06 (0.27)	0.85 (0.30)	p < 0.001
**Timed up and go (*sec*)**			8.02 (3.68)		10.10 (4.45)	16.10 (11.00)	p < 0.001
**Sit to stand (*sec*)**			10.27 (3.78)		11.49 (3.88)	15.40 (9.34)	p < 0.001
**Six-minute time trial (*sec*)**			466.09 (110.18)		379.24 (118.12)	291.28 (134.48)	p < 0.001
**EQVAS**			72.95(17.27)		63.53 (17.42)	55.40 (18.72)	p < 0.001
**EQ5D5L index score**			0.75 (0.26)		0.62 (0.26)	0.45 (0.34)	p < 0.001

At baseline, 62% of participants were robust, 23% prefrail, and 15% frail.

At baseline, increased frailty was associated with increased age; higher chronic condition count (X^2^(3)-23.036 < 0.001); poorer scores on each of gait speed (m/s), timed up and go (*sec*), five sit-to-stand, six-minute walk test (m), EQVAS, and EQ5D5L index scores; and with higher health service use as estimated by number of services attended.

Longitudinal data at 24 weeks was available for 725 participants. Change in mean scores for gait speed (m/s), timed up and go (*sec*), sit to stand (*sec*), six-minute walk test (m), EQVAS, and EQ5D5L index scores from induction to 24-week reassessment were analysed by Clinical Frailty Scale group and are presented in Table 2.

**Table 2:** Change in mean score for outcome measures from baseline to 24-week reassessment by Clinical Frailty Status group.

Outcome Measure	Clinical Frailty Scale Group	Induction	24 Week Reassessment	p value	n
***GST (m/s)***		*Value Mean (SD)*			
	Robust	1.24 (.242)	1.32 (.363)	<0.001	379
	Prefrail	1.05 (.267)	1.11 (.454)	0.135	124
	Frail	.89 (.037)	.97 (.044)	0.005	62
***TUG (sec)***		*Value Mean (SD)*			
	Robust	8.00(4.52)	7.51(3.50)	0.04	389
	Prefrail	10.03(4.56)	10.32(7.78)	0.53	126
	Frail	15.52(9.36)	13.52(10.08)	0.03	68
**Sit to Stand (*sec*)**		*Value Mean (SD)*			
	Robust	10.13(3.45)	8.90(3.80)	<0.001	380
	Prefrail	11.32(3.69)	9.81(2.87)	<0.001	122
	Frail	15.59(7.20)	14.21(7.38)	0.11	68
**Six-minute time trial (m)**		*Value Mean (SD)*			
	Robust	470.81(98.25)	521.16(128.34)	<0.001	386
	Prefrail	376.12(114.13)	400.55(131.27)	0.002	123
	Frail	295.72(123.65)	334.37(149.21)	0.002	68
**EQVAS**		*Value Mean (SD)*			
	Robust	75.44(16.58)	78.00(14.45)	0.004	376
	Prefrail	64.17(16.8)	67.76(15.92)	0.036	121
	Frail	56.47(18.95)	63.90(18.99)	0.004	71
**EQ5D5L Index Score**		*Value Mean (SD)*			
	Robust	.772(.219)	.794(.196)	0.01	351
	Prefrail	.626(.253)	.659(.245)	0.136	110
	Frail	.536(.286)	.561(.304)	0.46	65

For participants who were frail at baseline, there were statistically significant improvements in gait speed (m/s), time up and go (*sec*), six-minute time trial (m), and EQVAS. There were also improvements in the sit to stand test (*sec*) and EQ5D5L index score although not reaching statistical significance.

Prefrail participants had improvements in the sit to stand test (*sec*), six-minute time trial (m), and EQVAS. There were improvements in gait speed (m/s) and EQ5D5L index score, but these did not reach statistical significance. There was no improvement in timed up and go (*sec*).

The robust participants at baseline recorded improvements in all of gait speed (m/s), timed up and go (*sec*), sit to stand test (*sec*), six-minute walk test (m), EQVAS, and EQ5D5L index score.

Differences in the improvements observed differed significantly between the frailty groups for each outcome assessed other than the six-minute walk test. The frail group showed more improvement than the robust group in four of the five with significant differences and equal improvement in the other. (Supplementary Data Appendix F Table 1).

### 3.1 Change in gait speed frailty risk groups

Using gait speed as a proxy for frailty risk, 28% of participants improved their frailty risk group, 55% remained unchanged, and 17% disimproved (χ²(2) = 27.75, p < 0.001) (Supplementary Data Appendix F Table 5).

### 3.2 Attrition by frailty status

Programme dropout was explored by Clinical Frailty Scale group (Supplementary Data Appendix F Table 2).

The percentage of participants that dropped out of the program by 24 weeks increased with increasing frailty status (p < 0.002).

### 3.3 Regression analyses

Multivariable logistic regression (Supplementary Data Appendix F Table 3) was used to assess whether frailty status predicted program dropout, the model adjusted for age, comorbid condition count, and gender. Frail participants had significantly higher odds of program dropout than robust participants (OR 1.93 CI 1.35–2.78 p < 0.001).

Linear regression analysis (Supplementary Data Appendix F Table 4) showed that higher class attendance counts were associated with improved gait speed values at 24 weeks (β = 0.006 per class attended, CI 0.003–0.009, p = 0.001), after adjusting for baseline gait speed, frailty status, age, gender, and number of conditions.

## 4 Discussion

This secondary analysis of a prospective cohort study found a clinically meaningful positive association between participation in the ExWell programme with multiple physical outcome measures and quality of life in frail, prefrail, and robust community dwelling adults with chronic disease and multimorbidity, and with similar or greater gains among those who were frail compared to those who were robust at baseline. A significant body of evidence supports exercise therapy as an effective intervention for individuals living with prefrailty and frailty [6,7]. This study adds to this existing literature by providing real world evidence from a preexisting community based exercise rehabilitation programme It takes into account the complexities of delivering such a programme at scale in the community

There was significant representation of the frail and prefrail population in the study sample with 38% of participants classified as prefrail or frail. At baseline, increasing frailty (assessed using the Clinical Frailty Scale) was associated with a higher chronic condition count, with poorer scores on each of gait speed (m/s), timed up and go (*sec*), five sit-to-stand, six-minute walk test (m), EQVAS, and EQ5D5L index scores at baseline, and with higher health service use as estimated by number of healthcare services attended.

Frailty prevalence in the current study cohort was 15% and prefrailty was 23%, comparable with prevalence rates in the national longitudinal study of ageing (TILDA) of 11% with frailty and 44.5% with prefrailty [3], though TILDA participants are younger and may or may not suffer from chronic disease.

There is existing evidence that CBER programmes are effective in preventing and reversing prefrailty and frailty in older adults in trial settings [[6], [7], [8], [9], [10]]. The current study provides real-world data from an existing service that supports this evidence, demonstrating a significant longitudinal association with clinically meaningful improvements in frailty related outcomes, including objective physical tests and health related quality of life.

Lim et al.  concluded that CBER programs are effective in both improving gait speed and reversing pre-frailty [10]. The clinically significant improvement in gait speed of 0.08m/s in the current study cohort is consistent with previous studies [24,33,34] and was similar across all three frailty status groups (range: 0.06m/s-0.08m/s). A change in gait speed of 0.05m/s is associated with small meaningful clinical benefits and substantial benefits at 0.1m/s [24].

Significant improvements were also recorded in the timed up and go (TUG) test in both the robust and frail groups, but not in the prefrail group. TUG has been identified as a measure that can be utilised in frailty assessment, but not in assessing prefrailty [35]. The reduction of 2 s in the frail group is almost at the level of 2.1 s change that has been identified as a clinically meaningful change in functional status [36], and it brings the mean result for this group below 15 s, proposed as a cut off value for assessing falls risk [37]. The current study recorded a reduction of 1.4 s in the five time sit to stand test for frail participants post intervention; this reaches the threshold for minimum clinically important difference in older adults [38]. A 17.8 m [39] and 20 m [24] improvement in six minute walk test has been proposed as a clinically important change in this test in older adults; all three groups in the current study surpass both values.

We identified a significant improvement in health-related quality of life measured using the EQVAS for the robust (+3), prefrail (+4), and frail (+6) groups. This was below the MCID of 9 [40]; however, previous studies found no improvement in health-related quality of life in frail and prefrail older adults post exercise therapy so any improvement is encouraging [7,10]. Apostalo et al.  found that quality of life only improved with interventions that also included individually tailored care plans and psychological therapy [8].

Similar to Lim et al. we also found that increasing class attendance was associated with greater improvements in gait speed. After adjusting for baseline gait speed and other covariates, we found no evidence that the relationship between class attendance and gait speed differed by frailty group. These findings across studies may suggest that the frequency of exercise sessions may influence the effect size for gait speed [10] and highlights the importance of adherence to the exercise program. We identified that increasing frailty severity was associated with a higher rate of program dropout and lower class attendance. This may be an important area to focus on engaging and retaining more frail participants. Previous studies have reported that adherence to community exercise programs aimed at frail and older adults was high but this may vary with severity as found in our study [6,9,13]. A previous systematic review of the evidence for primary care interventions to delay and reverse frailty reported that a key differentiator in addressing participation in physical activity programs was ongoing encouragement from medical professionals [9].

Overall, a significant change in gait speed derived frailty risk status was observed between induction and 24-week reassessment. More participants improved than deteriorated, although some declines were also observed. This suggests a general shift towards improved frailty risk status following the intervention. A systematic review and meta-analysis of community exercise interventions in prefrail older adults concluded that individuals participating in community exercise had three times greater odds of reversing prefrailty to robust state [10]. Given that frailty prevalence increases [3] and severity progresses [26,27] with age, a net improvement of 11% of participants is encouraging.

## 5 Strengths and limitations

This study reports a secondary data analysis exploring frailty in a prospective cohort study evaluating a preexisting intervention running in community centres across Ireland, so its findings reflect real world implementation and the real-world logistical challenges involved in delivery of CBER programs. It was conducted and reported in line with the STROBE reporting guidelines [29].

The study design is a limitation as it can’t determine effectiveness, but randomisation of participants was not possible in this real-world setting. A further limitation relates to the lack of blinding of outcome assessors, which was not possible in this setting and within our study budget. However physical performance measures were relatively objective, minimising observer bias.

The substantial loss to follow up (44% of participants) increases the risk of attrition bias and may limit the generalizability of the results. However, measures were taken to contact those who were lost to follow up to explore reasons for disengagement and to invite them to attend for reassessment. Additional analyses exploring the impact of attrition were also reported in the main cohort paper indicating that observed improvements in the measures reported there are unlikely to be driven by attrition bias [28]

The use of the Clinical Frailty Scale to record baseline frailty status could be seen as a limitation. The Fried Frailty Criteria or the Frailty Index could have been utilised; however, the Clinical Frailty Scale was chosen as it is easy to administer in large numbers and has been shown to have comparative validity with the Frailty Index [41].

The lack of a multidimensional frailty measure at 24-week reassessment is a limitation but it was not possible to repeat the measure due to resource constraints. However, the use of gait speed as an objective proxy measure provided data on pre and post intervention frailty risk.

## 6 Conclusion

This study provided evidence that participation in the ExWell programme was associated with significant improvements in frailty risk across groups in community-dwelling adults living with one or more chronic conditions, outside the context of controlled trial settings. Programme completion by participants living with frailty was associated with improvements in physical performance and quality of life that mostly matched or exceeded those estimated for more robust participants, but attrition from the programme was also greater in participants living with frailty*.* Effective strategies to improve adherence and reduce attrition among adults living with pre-frailty and frailty in community-based exercise programmes warrant further investigation.

## Funding

This research project was supported by funding from the Irish Health Service Executive Community Healthcare Organisation 7 Primary Care Programme. The funder played no role in the design, execution, analysis and interpretation of data, or writing of the study.

## Declaration of generative AI and AI-assisted technologies in the writing process

AI and AI assisted technologies have not been used in the writing of this paper

## Data availability

The datasets generated and analysed as part of this research study are not currently publicly available.

## CRediT authorship contribution statement

**Muireann O’Shea:** Writing – review & editing, Writing – original draft, Visualization, Resources, Project administration, Methodology, Investigation, Formal analysis, Data curation, Conceptualization. **Paul Doody:** Writing – review & editing, Writing – original draft. **Noel McCaffrey:** Project administration, Methodology, Investigation, Funding acquisition, Conceptualization. **Aodhaghan O'Deasmhunaigh:** Project administration, Methodology, Investigation, Data curation, Conceptualization. **Fiona Skelly:** Methodology, Investigation, Conceptualization. **Lisa Kelly:** Writing – review & editing, Writing – original draft, Project administration. **Sean P Kennelly:** Writing – review & editing, Writing – original draft, Methodology, Conceptualization. **Susan M Smith:** Writing – review & editing, Writing – original draft, Visualization, Supervision, Project administration, Methodology, Funding acquisition, Formal analysis, Conceptualization. **Noel McCarthy:** Writing – review & editing, Writing – original draft, Visualization, Supervision, Project administration, Methodology, Formal analysis, Conceptualization.

## Declaration of competing interest

The authors declare the following financial interests/personal relationships which may be considered as potential competing interests:Muireann O Shea reports financial support was provided by Health Service Executive. If there are other authors, they declare that they have no known competing financial interests or personal relationships that could have appeared to influence the work reported in this paper.
