# Supplementary material for: Changes in physical performance and frailty-associated risk associated with participation in a community exercise programme: Findings from the ExWell cohort
Source: J Frailty Aging. 2026 Oct 5;15(5):100199. doi: 10.1016/j.tjfa.2026.100199 (PMC13671327; doi:10.1016/j.tjfa.2026.100199)
Supplement: Supplementary file 1 [file mmc1.docx]

Contents

[Appendix A STROBE Checklist	2](#_Toc228437807)

[Appendix B Physical Measurement Protocols	5](#_Toc228437808)

[1.	Physical function measures assessed	5](#_Toc228437809)

[2.	Testing protocol	6](#_Toc228437810)

[Appendix C Participant Flow	9](#_Toc228437811)

[Appendix D ExWell Intervention	10](#_Toc228437812)

[1.	Intervention description	10](#_Toc228437813)

[2.	TIdieR Checklist	13](#_Toc228437814)

[Appendix E	15](#_Toc228437815)

[Participant Questionnaire : Baseline	15](#_Toc228437816)

[Appendix F Supplementary Tables	20](#_Toc228437817)

[Table 1 Change in outcome measures from baseline to 24-week reassessment by Clinical Frailty Status with test of significance for difference between the frailty groups	20](#_Toc228437818)

[Table 2 Attrition rate by frailty status.	20](#_Toc228437819)

[Table 3 Results of logistic regression to assess whether frailty status predicted program dropout	20](#_Toc228437820)

[Table 4 Linear regression analysis of the impact of class attendance on gait speed adjusting for age, number of chronic conditions, gender, baseline gait speed, and frailty status	21](#_Toc228437821)

[Table 5 Change in gait speed frailty risk groups from induction to 24-week reassessment	22](#_Toc228437822)

# Appendix A STROBE Checklist

STROBE Statement—Checklist of items that should be included in reports of ***cohort studies***

|  | Item No | Recommendation |  | Page No. |  |
| --- | --- | --- | --- | --- | --- |
| **Title and abstract** | 1 | (*a*) Indicate the study’s design with a commonly used term in the title or the abstract |  | Page 2 |  |
|  |  | (*b*) Provide in the abstract an informative and balanced summary of what was done and what was found |  | Page 2 |  |
| Introduction | | |  |  |  |
| Background/rationale | 2 | Explain the scientific background and rationale for the investigation being reported |  | Page 3 |  |
| Objectives | 3 | State specific objectives, including any prespecified hypotheses |  | Page 4 |  |
| Methods | | |  |  |  |
| Study design | 4 | Present key elements of study design early in the paper |  | Page 4 |  |
| Setting | 5 | Describe the setting, locations, and relevant dates, including periods of recruitment, exposure, follow-up, and data collection |  | Pages 4,5 |  |
| Participants | 6 | (*a*) Give the eligibility criteria, and the sources and methods of selection of participants. Describe methods of follow-up |  | Page 4 |  |
|  |  | (*b*) For matched studies, give matching criteria and number of exposed and unexposed |  | NA |  |
| Variables | 7 | Clearly define all outcomes, exposures, predictors, potential confounders, and effect modifiers. Give diagnostic criteria, if applicable |  | Pages 5,6 |  |
| Data sources/ measurement | 8* | For each variable of interest, give sources of data and details of methods of assessment (measurement). Describe comparability of assessment methods if there is more than one group |  | Pages 5,6  Appendix B |  |
| Bias | 9 | Describe any efforts to address potential sources of bias |  | Page 6 |  |
| Study size | 10 | Explain how the study size was arrived at |  | Page 4 |  |
| Quantitative variables | 11 | Explain how quantitative variables were handled in the analyses. If applicable, describe which groupings were chosen and why |  | Page 6 |  |
| Statistical methods | 12 | (*a*) Describe all statistical methods, including those used to control for confounding |  | Page 6 |  |
|  |  | (*b*) Describe any methods used to examine subgroups and interactions |  | Page 6 |  |
|  |  | (*c*) Explain how missing data were addressed |  | Page 6 |  |
|  |  | (*d*) If applicable, explain how loss to follow-up was addressed |  | Pages 5, 6 |  |
|  |  | (*e*) Describe any sensitivity analyses |  | Page 6 |  |
| Results | | |  |  |  |
| Participants | 13* | (a) Report numbers of individuals at each stage of study—eg numbers potentially eligible, examined for eligibility, confirmed eligible, included in the study, completing follow-up, and analysed |  | Pages 6, 7 |  |
|  |  | (b) Give reasons for non-participation at each stage |  | Appendix 3.3 |  |
|  |  | (c) Consider use of a flow diagram |  | Page 7 |  |
| Descriptive data | 14* | (a) Give characteristics of study participants (eg demographic, clinical, social) and information on exposures and potential confounders |  | Page 7  Table 1 |  |
|  |  | (b) Indicate number of participants with missing data for each variable of interest |  | Tables 1, 2 |  |
|  |  | (c) Summarise follow-up time (eg, average and total amount) |  | Page 4 |  |
| Outcome data | 15* | Report numbers of outcome events or summary measures over time |  | Table 1, 2 |  |
| Main results | 16 | (*a*) Give unadjusted estimates and, if applicable, confounder-adjusted estimates and their precision (eg, 95% confidence interval). Make clear which confounders were adjusted for and why they were included |  | Page 10 |  |
|  |  | (*b*) Report category boundaries when continuous variables were categorized |  | NA |  |
|  |  | (*c*) If relevant, consider translating estimates of relative risk into absolute risk for a meaningful time period |  | NA |  |
| Other analyses | 17 | Report other analyses done—eg analyses of subgroups and interactions, and sensitivity analyses |  | Page 10 |  |
| Discussion | | |  |  |  |
| Key results | 18 | Summarise key results with reference to study objectives |  | Pages 10, 11 |  |
| Limitations | 19 | Discuss limitations of the study, taking into account sources of potential bias or imprecision. Discuss both direction and magnitude of any potential bias |  | Page 12 |  |
| Interpretation | 20 | Give a cautious overall interpretation of results considering objectives, limitations, multiplicity of analyses, results from similar studies, and other relevant evidence |  | Pages 11, 12 |  |
| Generalisability | 21 | Discuss the generalisability (external validity) of the study results |  | Pages 11, 12 |  |
| Other information | | |  |  |  |
| Funding | 22 | Give the source of funding and the role of the funders for the present study and, if applicable, for the original study on which the present article is based |  | Page 13 |  |

# Appendix B Physical Measurement Protocols

## 1. Physical function measures assessed

| Outcome Measure | Domain Assessed |
| --- | --- |
| Handgrip strength (kg) | Upper limb strength [1] |
| Timed five sit to stand and 10 sit to stand (sec) | Lower limb strength[2] |
| Six Minute Time Trial (m) | Functional exercise capacity[3] |
| Gait Speed test and gait speed test dual task (m/s) | Functional capacity, falls risk, frailty [4], cognitive function[4,5] |
| Timed up and go and timed up and go dual task (m/s) | Functional mobility[6], cognitive function[7], falls risk[8] |

1. Rijk JM, Roos PR, Deckx L *et al.* Prognostic value of handgrip strength in people aged 60 years and older: A systematic review and meta-analysis. *Geriatrics & Gerontology International* 2016;**16**:5–20.

2. Bohannon RW, Bubela DJ, Magasi SR *et al.* Sit-to-stand test: Performance and determinants across the age-span. *Isokinet Exerc Sci* 2010;**18**:235–40.

3. Singh SJ, Puhan MA, Andrianopoulos V *et al.* An official systematic review of the European Respiratory Society/American Thoracic Society: measurement properties of field walking tests in chronic respiratory disease. *European Respiratory Journal* 2014;**44**:1447–78.

4. Middleton A, Fritz SL, Lusardi M. Walking Speed: The Functional Vital Sign. *J Aging Phys Act* 2015;**23**:314–22.

5. Ramírez F, Gutiérrez M. Dual-Task Gait as a Predictive Tool for Cognitive Impairment in Older Adults: A Systematic Review. *Front Aging Neurosci* 2021;**13**:769462.

6. Podsiadlo D, Richardson S. The Timed “Up & Go”: A Test of Basic Functional Mobility for Frail Elderly Persons. *Journal of the American Geriatrics Society* 1991;**39**:142–8.

7. Åhman HB, Berglund L, Cedervall Y *et al.* Timed “Up & Go” Dual-Task Tests: Age- and Sex-Specific Reference Values and Test–Retest Reliability in Cognitively Healthy Controls. *Phys Ther* 2021;**101**:pzab179.

8. Hofheinz M, Schusterschitz C. Dual task interference in estimating the risk of falls and measuring change: a comparative, psychometric study of four measurements. *Clin Rehabil* 2010;**24**:831–42.

## 2. Testing protocol

*Dublin West Testing Protocol*

- ***All data to be entered to the excel on the day.*** *If you are taking results down on paper/notes, make sure to enter them on the excel before leaving the site.*
- *Responsibility of station lead to make sure all data for that station is captured*

***Height:***

- *Shoes off*
- *Back to the wall, facing out*
- *Measured in metres (for BMI formula)*

***Weight:***

- *Shoes off*
- *Measured in kg*

***Timed Up and Go:***

***Set up:***

- *Participant sitting in the chair*
- *Cone 3 metres away from chair*
- *Measure time in seconds*
- *1 trial – enter in excel*
- *Start clock as soon as you say go*
- *Stop clock when bum hits the seat again*

***Instruction to participant:***

- *‘When I say go, stand up and walk as quickly and as safely as you can around the cone and back and sit into the chair. Try not to use your arms when you stand up, but you can if you need to. The clock starts when I say go, and stops when you sit back down again’.*

***Timed up and Go Dual Task – Same set up***

***Dual Task Instructions:***

- *This time I want you to do the same task, but also to call out the months of the year in reverse order, for example December, November, October. The idea is to challenge the brain at the same time as walking and it might make it more difficult.’*

***6 Minute Walk Test***

***Set up:***

- *20m lengths*
- *Make sure to measure distance on last lap and write it on the sheet.*
- *Mark and calculate distance on sheet – transfer as final result to excel*

***Instruction to participant:***

- *‘This is the 6-minute time trial. In the 6 minutes, walk or run up and back as many times as you can (or combination of both) between this line and the line 20 metres away. Try and cover as much distance as you can in 6 minutes. The distance you cover is your score. You can rest if you like but should only rest if you need to. You should feel a little breathless, it’s an aerobic fitness test.* ***But****, if you feel any chest pain, dizziness or nausea, let us know immediately and we will stop the test. We will let you know when you have: 3 minutes left, 1 minute left, 30 seconds left, and 10 seconds left. When we say stop, stop exactly where you are.’*

***Handgrip:***

***Set up:***

- *Participant sitting in chair, shoulder adducted, elbow flexed to 90*
- *Put the dynamometer in their hand. Adjust the handle to fit their hand.*
- *Reset the needle to zero*
- *Tester can support the weight of dynamometer if necessary*
- *Observe - Grip should be applied smoothly, not jerking or wrenching. Repeat if not smooth.*
- *3 trials – enter each into excel*

***Instruction to participant:***

- *‘Squeeze the handle with maximum strength for 2 secs, then relax’*

***5 Time Sit-to-stand:***

***Set Up:***

- *Participant sitting in chair.*
- *2 trials – enter each into excel*
- *If best score is* ***less than 10 seconds****, complete also 1 trial of 10 time sit to stand*
- *If first trial is less than 10 seconds, don’t complete a second trial – go straight to 10 time sit to stand.*

***Instruction to participant:***

- *‘When I say go, stand up and sit back down 5 times as quickly as you can. I’ll be timing you on a stopwatch. Your time starts when your bum leaves the seat. Your time stops when you stand up for the 5^th^ time. Keep your hand across your chest if you can, but if you need to use your hands on the armrests or your knees, you can do that.’*

***10 Time Sit-to-Stand:***

- *Same instructions as 5 sit to stand except for 10 reps.*
- *Enter result into excel*

***Gait Speed Test:***

- *Cones at 0m – 1m – 5m – 6m*
- *Participant stands at 0m cone*
- *Start the timer with the first footfall after the 1m line.*
- *Stop the timer with the first footfall after the 5m line.*
- *1 trial only*
- *Enter result in ‘seconds’ into excel*

***Instruction to participant:***

- *‘When I say ‘Go’, Walk at a comfortable pace from where you are standing and stop at the last cone.’*

***Gait Speed Dual Task Test – Same set up***

***Dual Task Instructions:***

- *This time I want you to do the same task, but also to call out the months of the year in reverse order, for example December, November, October. The idea is to challenge the brain at the same time as walking and it might make it more difficult.’*

# Appendix C Participant Flow

*Figure 1 Participant flow from referral o 24-week reassessment*

** Not all participants who completed 24-week reassessment completed a 12-week reassessment also*

# Appendix D ExWell Intervention

## 1. Intervention description

**ExWell Intervention**

ExWell is a not-for-profit community-based organisation that delivers structured, group-based exercise programmes for adults living with chronic conditions. The programme operates under medical oversight, with classes delivered by exercise professionals certified by the British Association of Cardiovascular Prevention and Rehabilitation (BACPR), including individuals with backgrounds in physiotherapy, sports science and exercise physiology.

The programme consists of supervised group exercise sessions incorporating standardised elements: warm-up, aerobic training, resistance exercises, balance and core stability components, and cool-down. Participants include adults living with various chronic illnesses and multimorbdity who are referred to the programme by healthcare professionals or through social prescribing pathways. Prior to commencing classes, all participants attend a group induction session conducted by an ExWell physician or physiotherapist. This session includes orientation to the programme and baseline assessment of physical function and self-reported health outcomes. Participants are encouraged to attend two supervised sessions per week, with repeat assessment conducted at 12 weeks to monitor progress and outcomes.

**Materials**

There are no specific interventional materials. However, some resources are available on the ExWell website which offer guidance on exercising at home following the same principals within the in-person classes. <https://www.exwell.ie/_files/ugd/56bc1a_97afdf11228e436d913cfbed9f783bfc.pdf>

Equipment used in classes include dumbbells for strength workouts and in some centres, cardio machines such as exercise bikes, rowing machines and treadmills are available.

I**ntervention providers**

The induction sessions are delivered by a Medical Doctor who works with the ExWell programme.

Class delivery staff are qualified exercise professionals who have a range of different degrees such as physiotherapy, sports science, exercise physiology and personal training. ExWell delivery staff are also certified by the British Association of Cardiovascular Prevention and Rehabilitation (BACPR) and are trained in basic life support or cardiac first response.

**Location**

The intervention takes place in various community halls. Each centre is wheelchair accessible and has suitable parking for participants.

Each hall is equipped with chairs to accommodate chair-based classes, dumbbells for the strength component of classes and in some centre’s cardiovascular machines like rowers, exercise bikes and treadmills.

**Intervention Delivery**

The intervention is embedded into the real-world delivery schedule of the ExWell Medical Programme across Dublin West and Kildare based centres.

The exercise intervention involves twice weekly classes. Each class lasts 45 minutes in duration, comprising a warmup (5 mins), aerobic exercise (15 mins), strength work (10 mins), core stability and balance (10 mins), and a cool-down (5 mins). The standard class structure is divided into stations which allows participants to be assigned to groups of similar functional ability.

Examples of exercises included in a session are outlined in the following document from page 13 to 34 <https://www.exwell.ie/_files/ugd/56bc1a_97afdf11228e436d913cfbed9f783bfc.pdf>

**Warm-up content**

The warm-up consists of exercises that gently raise the heart rate and some dynamic stretches to get the body ready for the rest of the class. The warm-up starts at a low intensity and builds to a higher intensity towards the end.

Examples of warm-up content:

Marching on the spot, heel taps, side jacks, boxing, jogging on the spot, knee raise.

Arm swings, shoulder rotations, hip rotations, side bend, leg swings, hamstring sweeps, ankle rotations.

**Aerobic station**

For the aerobics section, participants are instructed to work at a moderate level of intensity. The guide used at classes is the talk test. Participants are informed that while exercising, they should still be able to talk but should not be able to sing. They should feel a little breathless and should be sweating a little and have a flushed face.

For each exercise, three levels are demonstrated to accommodate different ability levels. For example, jumping jacks.

Level 1: participants could sit in a chair and alternate tapping one leg out to the side with the same arm reaching over head.

Level 2: standing behind a chair participants could hold the back of the chair for balance if needed and alternated tapping the leg out to the side and reaching the same side arm overhead.

Level 3: full jumping jacks jumping both feet out with arms reaching up then jumping both feet in with arms coming back down.

An example of the structure of an aerobic session includes focusing on 3 rounds of 4 exercises. The participants would be guided to work at a moderate level of intensity for 40 seconds and then rest for 20 seconds. A one-minute break would be included in between each round to allow participants to sip on water as needed.

**Strength station**

At the strength station, participants could choose the weight of dumbbells that they felt able for. They were encouraged to choose a weight that would feel difficult for the last 2 to 3 reps when completing 10 to 12 reps.

The strength components targeted main muscles groups of the upper and lower body.

Example of exercises included: squats, deadlift, heel raises, lunges, side lunges, shoulder press, bicep curls, lateral and frontal raises, bent over row etc.

Different options were demonstrated for those who had mobility impairments.

**Core stability and balance exercises**

Core stability exercises were generally done sitting on a chair. They included exercises like leg raises, abdominal crunches, leg flutters.

Balance exercises were generally performed beside a wall or behind a chair so that there was something to hold onto as needed. Exercises included single leg holds, toe reaches in different planes of motion, tandem stance, tandem stance walks, lateral leg raises etc.

**Cooldown**

The cooldown focused on safely lowering the heart rate. Gently moving on the spot and finishing with static stretching of major muscles groups.

Throughout the classes, participants with specific mobility challenges received appropriate guidance and alternative instructions from staff members.

## 2. TIdieR Checklist

**The TIDieR (Template for Intervention Description and Replication) Checklist*:**

 Information to include when describing an intervention and the location of the information

| **Item number** | **Item** | **Where located **** | |
| --- | --- | --- | --- |
|  |  | PPrimary paper  ppage or appendix  nnumber) | Other ^†^ (details) |
|  | **BRIEF NAME** |  |  |
| **1.** | Provide the name or a phrase that describes the intervention. | Page 1 | Abstract, ‘Introduction’ |
|  | **WHY** |  |  |
| **2.** | Describe any rationale, theory, or goal of the elements essential to the intervention. | Page 3 | Methods, ‘ExWell Intervention’ |
|  | **WHAT** |  |  |
| **3.** | Materials: Describe any physical or informational materials used in the intervention, including those provided to participants or used in intervention delivery or in training of intervention providers. Provide information on where the materials can be accessed (e.g. online appendix, URL). | Supplementary Material Appendix D | Supplementary Material Appendix D |
| **4.** | Procedures: Describe each of the procedures, activities, and/or processes used in the intervention, including any enabling or support activities. | Page 3 | Methods, ‘ExWell Intervention’  Supplementary Material Appendix D |
|  | **WHO PROVIDED** |  |  |
| **5.** | For each category of intervention provider (e.g. psychologist, nursing assistant), describe their expertise, background and any specific training given. | Supplementary Material Appendix D | Supplementary Material Appendix D |
|  | **HOW** |  |  |
| **6.** | Describe the modes of delivery (e.g. face-to-face or by some other mechanism, such as internet or telephone) of the intervention and whether it was provided individually or in a group. | Page 2 | Methods, ‘Study Design’  Supplementary Material Appendix D |
|  | **WHERE** |  |  |
| **7.** | Describe the type(s) of location(s) where the intervention occurred, including any necessary infrastructure or relevant features. | Page 3 | Methods, ‘ExWell Intervention’  Supplementary Material Appendix 4 |
|  | **WHEN and HOW MUCH** |  |  |
| **8.** | Describe the number of times the intervention was delivered and over what period of time including the number of sessions, their schedule, and their duration, intensity or dose. | Supplementary Material Appendix D | Supplementary Material Appendix D |
|  | **TAILORING** |  |  |
| **9.** | If the intervention was planned to be personalised, titrated or adapted, then describe what, why, when, and how. | Page 3 | Methods, ‘ExWell Intervention’  Supplementary Material Appendix D |
|  | **MODIFICATIONS** |  |  |
| **10.^ǂ^** | If the intervention was modified during the course of the study, describe the changes (what, why, when, and how). | NA. The intervention was not modified during the course of the study. |  |
|  | **HOW WELL** |  |  |
| **11.** | Planned: If intervention adherence or fidelity was assessed, describe how and by whom, and if any strategies were used to maintain or improve fidelity, describe them. | Page 4 | Methods ‘Data Collection’ |
| **12.^ǂ^** | Actual: If intervention adherence or fidelity was assessed, describe the extent to which the intervention was delivered as planned. | Page 9 | Results ‘Class Attendance’  Supplementary Data Appendix F Table 2 |

# Appendix E

## Participant Questionnaire : Baseline

Medically Supervised Exercise Rehabilitation Program For Individuals with Chronic Disease: A Feasibility Study Exploring Processes and Impact of the ExWell Program

**STUDY ID:**

Demographics

Name:

DOB:

Address:

Contact number:

Marital Status:

**STUDY ID:**

Healthcare Details

**Details of your GP:**

GP Name	GP Location

**Who referred you to the ExWell Program:**

Name of person who referred you to ExWell­­	_____________________________

**Name of any medical services you attend. Please tick all that apply.**

| Hospital Consultant |  | Chiropodist |  |
| --- | --- | --- | --- |
| Physiotherapist |  | Psychology |  |
| Occupational Therapist |  | Home Help |  |
| Speech and Language Therapist |  | Meals on Wheels |  |
| Dietitian |  | Day Centre Services |  |
| Public Health Nurse |  | Optician |  |

Chronic conditions:

Has a doctor ever told you that you have any of the below conditions? Please tick all that apply.

|  | **Tick All that Apply** |  | **Tick All That Apply** |
| --- | --- | --- | --- |
| ***Lung*** | | ***Cancer*** | |
| Chronic lung disease such as chronic bronchitis, emphysema, COPD |  | Cancer or a malignant tumour |  |
| Asthma |  |  | |
| ***Neurological*** | | ***Eyes*** |  |
| Parkinson's Disease |  | Cataracts |  |
| Dementia, organic brain syndrome, senility |  | Glaucoma |  |
| Serious memory impairment |  | Age related macular degeneration |  |
| Alzheimer's Disease |  |  | |
| Epilepsy |  |  | |
| ***General Medical*** | | ***Heart*** | |
| Thyroid problems |  | High blood pressure or hypertension |  |
| Cirrhosis or serious liver damage |  | Angina |  |
| Stomach Ulcers |  | Atrial fibrillation |  |
| Chronic Kidney Disease |  | An abnormal heart rhythm (not atrial fibrillation) |  |
| Severe Anaemia |  | Diabetes or high blood sugar |  |
| ***Mental Health*** | | A stroke (cerebrovascular disease |  |
| Any emotional, nervous or psychiatric problems, such as depression or anxiety |  | Mini stroke or TIA |  |
| ***Miscellaneous*** | | High cholesterol |  |
| Substance Abuse |  | A heart murmur |  |
| Alcohol abuse |  | A heart attack (including myocardial infarction or coronary thrombosis) |  |
| Varicose Ulcers |  | Congestive Heart Failure |  |
| ***Musculoskeletal*** | | | |
| Arthritis (including osteoarthritis, rheumatoid arthritis) |  |  | |
| Osteoporosis |  |  | |
| ***Any other medical problems not mentioned above, please specify:*** | | | |

Health Behaviours

Do you currently smoke or have you ever smoked? Please tick the box that applies to you.

| Current Smoker |  |
| --- | --- |
| Ex Smoker |  |
| Never smoker |  |

Regarding alcohol, please tick the boxes that apply to you.

| **How often do you have a drink containing alcohol?** | Never | Less than monthly | 2-4 times per month | 2-3 times per week | 4 or more times per week |
| --- | --- | --- | --- | --- | --- |
| **How many drinks containing alcohol do**  **you have on a typical day when you drink?** | 1 or 2 drinks | 3-4 drinks | 5 or 6 drinks | 7-9 drinks | 10 or more drinks |
| **How often do you have six or more drinks on one occasion?** | Never | Less than monthly | Monthly | Weekly | Daily or almost daily |

Overall, in the past week, on how many days have you done a total of 30 minutes or more of physical activity, which was enough to raise your breathing rate?

This may include sport, exercise, and brisk walking or cycling for recreation or to get to and from places but should not include housework or physical activity that may be part of your job. Please circle the relevant number.

| 0. | 1. | 2. | 3. | 4. | 5. | 6. | 7. |
| --- | --- | --- | --- | --- | --- | --- | --- |

**Fear of Falling**

1. Are you afraid of falling? Y/N
2. If yes, does fear of falling cause you to limit your activities? Y/N

# Appendix F Supplementary Tables

## Table 1 Change in outcome measures from baseline to 24-week reassessment by Clinical Frailty Status with test of significance for difference between the frailty groups

| **Outcome Measure** | **Change from baseline to 24 weeks (mean, SD)** | | | | **p value** | ***n*** |
| --- | --- | --- | --- | --- | --- | --- |
|  | **Robust** | **Prefrail** | **Frail** | **All participants** |  |  |
| **Gait speed (m/s)** | 0.08 (0.34) | 0.06 (0.41) | 0.08 (0.23) | 0.08(0.35) | p<0.001 | 564 |
| **Timed up and go (sec)** | -0.38(4.62) | 0.29 (5.19) | -2.01 (7.19) | -0.49(5.13) | p<0.001 | 582 |
| **Sit to stand (sec)** | -1.22(3.50) | -1.51 (3.10) | -1.44 (7.01) | -1.31(3.99) | p<0.001 | 569 |
| **Six-minute walk test (m)** | 50.35(17.26) | 24.43(83.44) | 38.25(98.81) | 43.41(91.02) | p=0.266 | 576 |
| **EQVAS** | 2.56(17.26) | 3.59)18.61) | 7.69(21.35) | 3.41(18.14) | p=0.049 | 567 |
| **EQ5D5L index score** | 0.02(0.18) | 0.03(0.23) | 0.03(0.28) | 0.02(0.20) | p<0.001 | 525 |

## Table 2 Attrition rate by frailty status.

| **Clinical Frailty Status Group** | **N** | **Stayed** | **Lost to follow up** |
| --- | --- | --- | --- |
| **Robust** | 668 | 380 (57%) | 288 (43%) |
| **Prefrail** | 250 | 123 (49%) | 127 (51%) |
| **Frail** | 166 | 71 (43%) | 95 (57%) |
| **Total** | 1084 | 574 (53%) | 510 (47%) |
| **P value** | 0.002 |  |  |

## Table 3 Results of logistic regression to assess whether frailty status predicted program dropout

| **Predictor** | **Odds Ratio** | **95% CI** | **p value** |
| --- | --- | --- | --- |
| **Frailty Status** |  |  |  |
| Prefrail v Robust | 1.35 | 1.00-1.84 | 0.05 |
| Frail v Robust | 1.93 | 1.35-2.78 | <0.001 |
| Age | 0.96 | 0.95-0.98 | <0.001 |
| No of chronic conditions | 1.09 | 1.03-1.16 | 0.004 |
| Gender |  |  |  |
| Male | 1.09 | 0.85-1.41 | 0.5 |
| n | 1049 |  |  |
| p value | <0.001 |  |  |
| PseudoR^2^ | 0.04 |  |  |

## Table 4 Linear regression analysis of the impact of class attendance on gait speed adjusting for age, number of chronic conditions, gender, baseline gait speed, and frailty status

| **Predictor** | **B coefficient** | **95% CI** | **p value** |
| --- | --- | --- | --- |
| Classes attended | 0.006 | 0.003-0.009 | 0.001 |
| Frailty Status |  |  |  |
| Prefrail v Robust | -0.097 | -0.266-0.072 | 0.261 |
| Frail v Robust | -0.186 | -0.416-0.044 | 0.112 |
| Prefrail v classcount | 0.001 | -0.006-0.007 | 0.812 |
| Frail v classcount | 0.004 | -0.015-0.014 | 0..358 |
| Baseline gait speed | 0.692 | 0.578-0.806 | <0.001 |
| Age | -0.004 | -0.007--0.0004 | 0.029 |
| Condition Count | 0.006 | -0.008-0.020 | 0.397 |
| Gender | -0.019 | -0.078-0.040 | 0.529 |
| n | 546 |  |  |
| R^2^ | 0.33 |  |  |
| Adjusted R^2^ | 0.32 |  |  |
| F statistic | 29.6 |  |  |
| p value | <0.001 |  |  |

## Table 5 Change in gait speed frailty risk groups from induction to 24-week reassessment

|  |  |  | **Reduced by 2** | **Reduced by 1** | **No Change** | **Improved by 1** | **Improved by 2** | **Total** |
| --- | --- | --- | --- | --- | --- | --- | --- | --- |
| **CFS Grouped** | **Robust** | Count | 26 | 34 | 222 | 81 | 16 | 379 |
|  |  | % | 6.8% | 8.9% | 58.5% | 21.3% | 4.2% | 100% |
|  | **Prefrail** | Count | 11 | 20 | 55 | 30 | 10 | 126 |
|  |  | % | 8.7% | 15.8% | 43.6% | 23.8% | 7.9% | 100% |
|  | **Frail** | Count | 1 | 5 | 34 | 18 | 5 | 63 |
|  |  | % | 1.5% | 7.9% | 53.9% | 28.5% | 7.9% | 100% |
|  | **Total** | Count | 38 | 59 | 311 | 129 | 31 | 568 |
|  |  | % | 6.7% | 10.4% | 54.8% | 22.7% | 5.4% |  |

The difference in gait speed frailty risk groups between induction and 24-week reassessment was assessed and is presented in the below above. Reduced by 2 indicates that participants went down 2 categories (eg from Robust to Frail), similarly reduced by 1 indicates participants went down 1 category (eg from Prefrail to Frail). The same logic is applied to those improving by 1 or 2.

The change in gait speed frailty risk group from induction to 24-week reassessment was cross tabulated by Clinical Frailt Status group to explore the transition of participants between gait speed categories by Clinical Frailty Status.
